# Cyclin-Dependent Kinase 9 Activity Regulates Neutrophil Spontaneous Apoptosis

**DOI:** 10.1371/journal.pone.0030128

**Published:** 2012-01-19

**Authors:** Keqing Wang, Peter Hampson, Jon Hazeldine, Vladimir Krystof, Miroslav Strnad, Paul Pechan, Janet M.

**Affiliations:** 1 MRC Centre for Immune Regulation, Institute of Biomedical Research, Birmingham University Medical School, Birmingham, United Kingdom; 2 Laboratory of Growth Regulators, Institute of Experimental Botany ASCR and Palacký University, Olomouc, Czech Republic; 3 C3Bio Ltd, Munich, Germany; French National Centre for Scientific Research - Université Aix-Marseille, France

## Abstract

Neutrophils are the most abundant leukocyte and play a central role in the immune defense against rapidly dividing bacteria. However, they are also the shortest lived cell in the blood with a lifespan in the circulation of 5.4 days. The mechanisms underlying their short lifespan and spontaneous entry into apoptosis are poorly understood. Recently, the broad range cyclin-dependent kinase (CDK) inhibitor R-roscovitine was shown to increase neutrophil apoptosis, implicating CDKs in the regulation of neutrophil lifespan. To determine which CDKs were involved in regulating neutrophil lifespan we first examined CDK expression in human neutrophils and found that only three CDKs: CDK5, CDK7 and CDK9 were expressed in these cells. The use of CDK inhibitors with differing selectivity towards the various CDKs suggested that CDK9 activity regulates neutrophil lifespan. Furthermore CDK9 activity and the expression of its activating partner cyclin T1 both declined as neutrophils aged and entered apoptosis spontaneously. CDK9 is a component of the P-TEFb complex involved in transcriptional regulation and its inhibition will preferentially affect proteins with short half-lives. Treatment of neutrophils with flavopiridol, a potent CDK9 inhibitor, increased apoptosis and caused a rapid decline in the level of the anti-apoptotic protein Mcl-1, whilst Bcl2A was unaffected. We propose that CDK9 activity is a key regulator of neutrophil lifespan, preventing apoptosis by maintaining levels of short lived anti-apoptotic proteins such as Mcl-1. Furthermore, as inappropriate inhibition of neutrophil apoptosis contributes to chronic inflammatory diseases such as Rheumatoid Arthritis, CDK9 represents a novel therapeutic target in such diseases.

## Introduction

Neutrophils are the shortest-lived and most abundant leukocytes, dying by apoptosis within 5.4 days of leaving the bone marrow [1]. They form part of the immune system's first line of defence against rapidly dividing bacteria and their functional lifespan can be extended at sites of infection via the anti-apoptotic actions of pro-inflammatory cytokines, such as GM-CSF [2]. This process is tightly regulated in order to avoid inappropriate survival of neutrophils which can lead to chronic inflammatory diseases such as Rheumatoid arthritis [3]. Despite the key role these cells play in innate immunity and chronic inflammatory disease, our understanding of the processes that regulate their lifespan remains incomplete. It has been established that levels of the anti-apoptotic protein Mcl-1 decline as neutrophils age and enter apoptosis [4] and factors that extend neutrophil lifespan, such as GM-CSF, act by increasing expression of Mcl-1 [5]. Determining the primary cause of loss of key neutrophil Bcl-2 family proteins such as Mcl-1 is thus central to understanding the short lifespan of neutrophils.

Rossi *et al* reported the surprising observation that the broad range cyclin-dependent kinase (CDK) inhibitor R-roscovitine increased the apoptosis of neutrophils [6], which are non-proliferating cells. R-roscovitine treatment also accelerated the loss of Mcl-1. The cellular target of roscovitine was suggested to be the cell cycle related cyclin-dependent kinases CDK1 or CDK2 [6]. However, expression of cell cycle related CDKs is lost as myeloblasts differentiate towards mature neutrophils [7], suggesting that these CDKs are unlikely to mediate the pro-apoptotic effects of roscovitine. Crucially, this publication did not consider the involvement of the cell cycle independent CDKs and to our knowledge CDK1/2 have not been implicated in processes other than cell cycle regulation. More recently the same group investigated possible non-CDK targets of R-roscovitine, but excluded a role for off-target inhibition of MAP kinase or NF-κB signalling [8].

We therefore reconsidered the role of CDKs in regulating neutrophil apoptosis and Mcl-1 expression and our findings suggest that a cell cycle independent CDK, CDK9, is in fact a key regulator of neutrophil apoptosis and lifespan.

## Results

**Figure 1:**
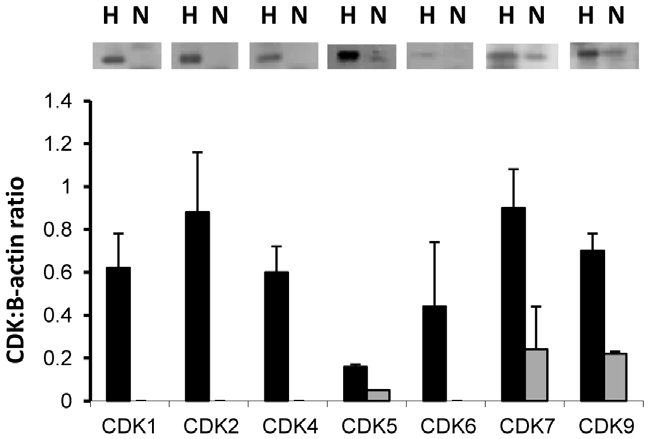
Human neutrophils express only cell cycle independent CDKs. Isolated human neutrophils (N) and promyelocytic HL60 cells (H) were assessed for expression of CDK proteins by western blotting (upper panel). Blots were scanned to determine the CDK:β-actin ratio by densitometry (lower panel; black bars  =  HL60, grey bars  =  neutrophils). Data are mean ± s.d. for three separate cell extracts.

We first determined the expression of CDKs in human neutrophils and found that only three were readily detected by western blotting (Fig. 1), namely the cell cycle-independent CDKs: CDK5, CDK7 and CDK9. Of these, CDK 7 and CDK9 were the predominant CDKs present, with CDK5 present only at a very low level. The promyelocytic cell line HL60 was used as a positive control for CDK expression. We could not detect any of the cell cycle-dependent CDKs (CDK1, CDK2, CDK4 or CDK6) in neutrophils, as would be expected of non-cycling cells, though all were expressed in the proliferating promyeloid HL60 cells. This is in broad agreement with previous reports showing that promyeloid progenitor cells lose expression of cell cycle dependent CDKs as they mature and differentiate towards neutrophils [7].

**Figure 2:**
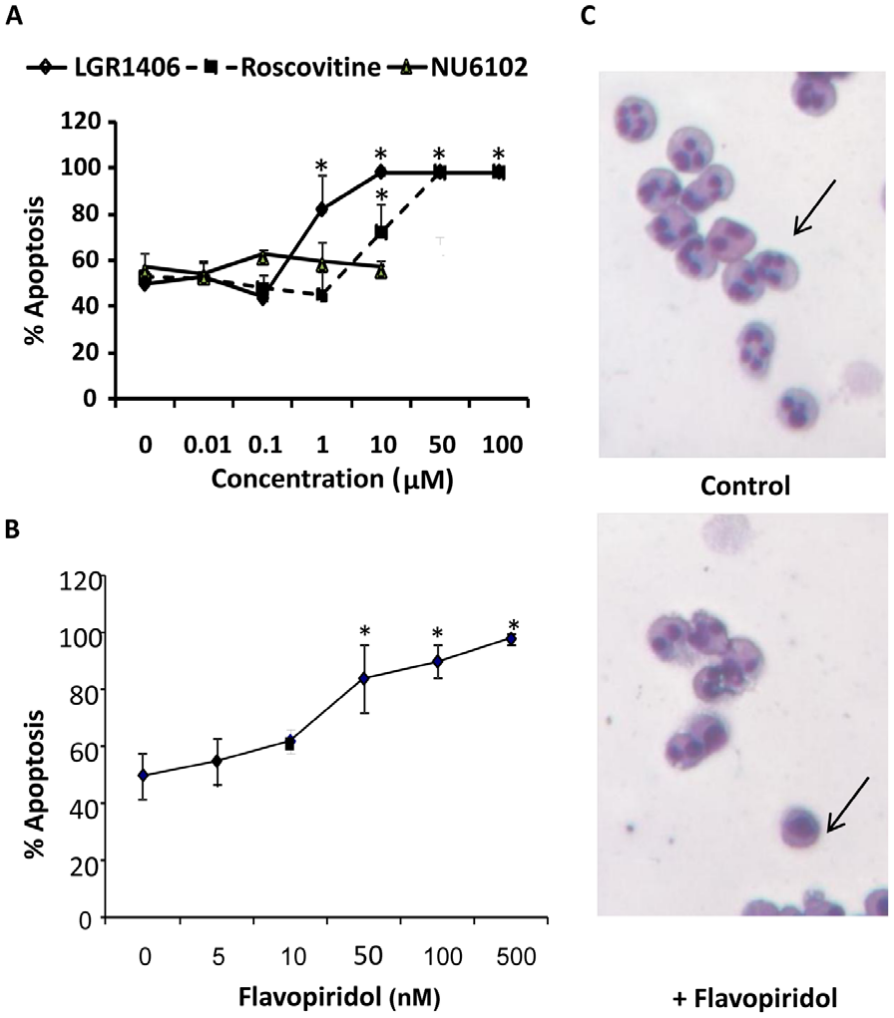
Inhibition of CDK9 but not cell cycle dependent CDKs increases neutrophil apoptosis. (a) Isolated human neutrophils were incubated with NU6102 (filled triangle), R-roscovitine (filled square), or LGR1406 (filled diamond) at the concentrations shown. Apoptosis was determined after 15h by assessment of DiOC_6_ uptake and flow cytometry, with DiOC_6_ low cells taken as apoptotic. Data are mean ± s.d. for four separate experiments. * indicates p<0.05 for treatment versus control. (b) Neutrophils were incubated with flavopiridol for 15 h at the concentrations shown and apoptosis determined by assessment of DiOC_6_ uptake. Data are mean ± s.d. for three separate experiments. * indicates p<0.05 for control versus flavopiridol treated cells. (c) Images of Giemsa stained neutrophils cultured in the absence (control) or presence of 100 nM flavopiridol for 15 h.

We then attempted to confirm the published effects of the broad range CDK inhibitor R-roscovitine [6], [8], showing that this compound increased neutrophil spontaneous apoptosis in a concentration dependent manner (Fig. 2a). We also assessed apoptosis using Annexin V binding and propidium iodide staining and confirmed that cells were not induced to enter necrosis during this time frame (data not shown). The short lifespan of neutrophils in culture (1–2 days) means that they are not amenable to knockdown strategies such as transfection with siRNA. In order to determine which of the three CDKs expressed by neutrophils were involved in the regulation of apoptosis we used pharmacological CDK inhibitors with appropriate selectivity profiles. A selective inhibitor of CDK1 and CDK2, NU6102 (IC_50_ 9 nM and 6 nM, respectively) [9], which does not inhibit transcriptional CDKs, (CDK7 and CDK9) in whole cells [10], had no effect on neutrophil apoptosis (Fig. 2a **)**. NU6102 also has some activity towards CDK5, with an IC_50_ of 0.48 µM (Lan-Zhen Wang, Newcastle University, personal communication), suggesting that the transcriptional CDKs are mediating the effects of roscovitine on neutrophil apoptosis. In contrast, a novel roscovitine analogue LGR 1406 which does not inhibit CDK7 [11] was able to increase neutrophil apoptosis (Fig. 2a). To further investigate the possible role of CDK9 we examined the effects of flavopiridol, a potent inhibitor of CDK9 [12], on neutrophil apoptosis. Flavopiridol was the most effective at accelerating entry of neutrophils in to apoptosis (Fig. 2b) and flavopiridol treated neutrophils showed the collapsed nuclear morphology characteristic of apoptotic neutrophils (Fig. 2c).

**Figure 3:**
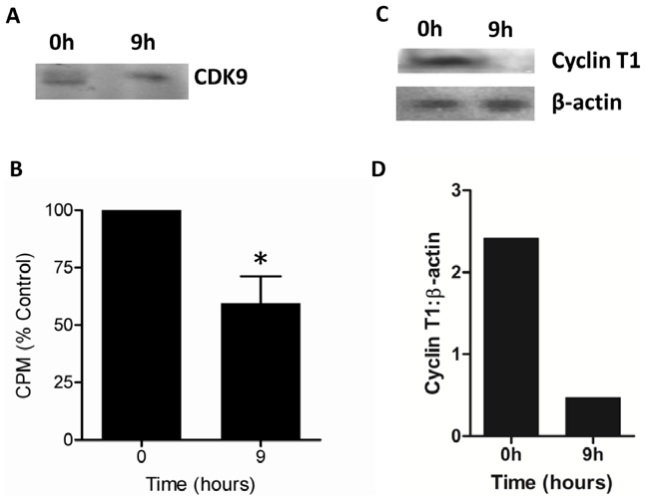
CDK9 activity and cyclin T1 expression decrease as neutrophils age in culture. Isolated human neutrophils were cultured for 9h and (a) CDK9 protein expression and (b) enzymatic activity were determined and compared with freshly isolated cells (0h). For CDK9 activity protein was immunoprecipitated from neutrophils with a monoclonal anti-CDK9 antibody and activity determined by incorporation of [γ ^32^P]-ATP into the CTD of RNA polymerase II. The phosphorylated peptide was isolated on an SDS-PAGE gel and excised for scintillation counting. Data are expressed as % of the value for control freshly isolated cells and are mean ± s.d. for three separate experiments. * indicates p<0.05. (c). Isolated neutrophils were cultured for 0 h or 9 h and expression of cyclin T1 determined by western blotting. Blots were probed with an antibody to β-actin to confirm equal protein loading. The blot shown is representative of 4 separate experiments performed. (d) Western blots of CDK9 expression relative to β-actin were quantitated using densitometry.

Upon association with its activating partner cyclin T1, CDK9 forms P-TEFb, a general transcription factor responsible for phosphorylating the C-terminal domain (CTD) of RNA polymerase II [13]. If CDK9 activity determines neutrophil lifespan we hypothesised that its activity and/or expression should decline as neutrophils aged prior to their entry into apoptosis. We therefore determined CDK9 expression and activity in freshly isolated neutrophils and after 9h in culture, at which time point less than 10% of neutrophils will have entered apoptosis. Figure 3a shows that expression of CDK9 was maintained as neutrophils aged, but CDK9 activity decreased dramatically prior to their entry into apoptosis (Fig. 3b). We further investigated the loss of CDK9 activity by determining expression of cyclin T1 and found that its expression was dramatically reduced by 9h in culture (Fig. 3c and 3d).

**Figure 4:**
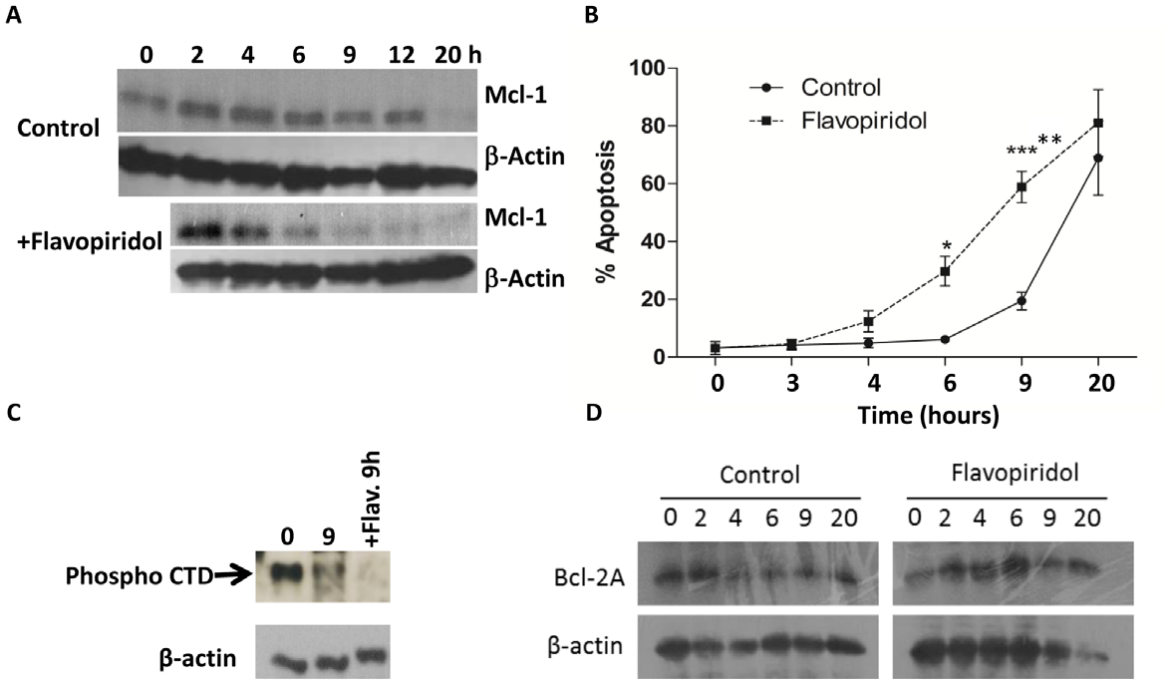
Flavopiridol treatment accelerates loss of Mcl-1. (a) Isolated human neutrophils were cultured in the absence (control) or presence of 100 nM flavopiridol for 0-20 h and Mcl-1 protein expression measured by western blotting. Blots were probed for β-actin to confirm equal loading. (b) In the same experiments neutrophil apoptosis was determined by measurement of DiOC_6_ uptake. Data are expressed as the % of apoptotic cells in the control (solid line) and flavopiridol (broken line) treated cultures and are mean ± s.d. for four separate experiments. * indicates p<0.05 and ** indicates p<0.01 for control versus flavopiridol treated cells. (c) CDK9 activity was measured in control, flavopiridol (100 nM) and flavopiridol plus GM-CSF (50 ng/ml) treated neutrophils by immunoprecipitating CDK9 from neutrophils with a monoclonal anti-CDK9 antibody and determining activity by assessing incorporation of [γ ^32^P]-ATP into the CTD of RNA polymerase II. The phosphorylated peptide was isolated on an SDS-PAGE gel and visualised by autoradiography. The image shown is representative of 3 separate experiments performed. (d) Isolated human neutrophils were cultured in the absence (control) or presence of 100 nM flavopiridol for 0–20 h and Bcl2A protein expression measured by western blotting. Blots were probed for β-actin to confirm equal loading.

As CDK9 is a regulator of transcription inhibition of CDK9 activity will have the most significant effect on the expression of proteins with a short half life. In the case of neutrophils this would include the key anti-apoptotic protein Mcl-1. Figure 4 shows that up to 6h post isolation the protein level of Mcl-1 protein remained stable, but by 9h the level of Mcl-1 began to decline and was only 20% of the control level by 20h in culture. Inhibition of CDK9 with flavopiridol accelerated the loss of Mcl-1 protein (Fig. 4a), confirming reports of the effects of flavopiridol on Mcl-1 mRNA levels [14], [15]. Furthermore, loss of Mcl-1 protein correlated with the increased neutrophil apoptosis seen over the same time scale (Fig. 4b). Measurement of CDK9 enzymatic activity confirmed that flavopiridol had inhibited the enzyme in neutrophils (Fig. 4c). To determine if other Bcl-2 family proteins expressed in neutrophils were also affected by CDK9 inhibition we determined expression of Bcl2A during in vitro culture and found that its expression did not change during a 20h period. (Fig. 4d). In addition inhibition of CDK9 with flavopiridol did not result in loss of Bcl2A (Figure 4d).

**Figure 5:**
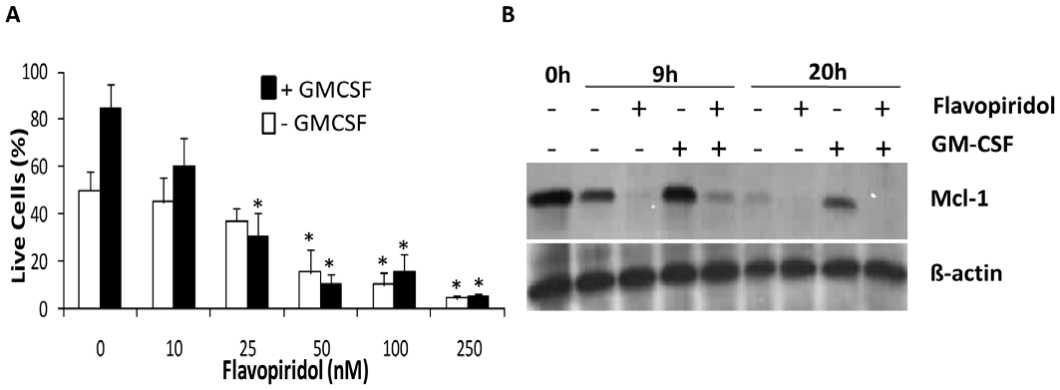
Flavopiridol inhibits upregulation of Mcl-1 by GM-CSF. (a). Neutrophils were incubated with medium alone, or medium containing 100 nM flavopiridol in the absence (open bars) or presence of (filled bars) of 50 ng/ml GM-CSF. Live cells were detected by their ability to retain DiOC_6_ and data are mean ± s.d. of 3 separate experiments. * indicates p<0.05 for control versus flavopiridol treated cells. (b). The same cells were extracted and measured for Mcl-1 content by western blotting. Blots were probed for β-actin to confirm equal loading.

Neutrophils receive survival signals at sites of inflammation from cytokines such as GM-CSF and type 1 interferons. These cytokines mediate their effects via upregulation of survival genes such as Mcl-1 and Bcl-XL [16], [17]. We therefore assessed the effects of flavopiridol on neutrophil apoptosis in the presence of GM-CSF. Figure 5a shows that flavopiridol induced neutrophil apoptosis even in the presence of GM-CSF and was able to prevent the upregulation of Mcl-1 protein by GM-CSF (Fig. 5b). Unsurprisingly then GM-CSF was also not able to overcome the inhibition of CDK9 activity by flavopiridol (Figure 4c).

Taken together these results identify CDK9 as a key regulator of the expression of Mcl-1, and thus of neutrophil apoptosis and lifespan.

## Discussion

Neutrophils form the first line of defence against pathogens such as rapidly dividing bacteria and fungi. They are produced by the bone marrow in vast numbers, approximately 10^11^ are released into the circulation each day and this can more than double at times of infection. This significant endeavour is due in great part to the very short lifespan of neutrophils, which die by apoptosis within approximately 5 days of their production [1]. Although the short lifespan of neutrophils has been known for over 30 years, the initiating event leading to the entry of neutrophils into apoptosis has not been defined. The work of the Edwards group and others [5], [6], [18] has shown that the bcl-2 family protein Mcl-1 is lost as neutrophils enter apoptosis and that factors that delay neutrophil apoptosis act via increasing or maintaining expression of Mcl-1, though other Bcl-2 family proteins also respond to survival factors including Bcl-XL [19]. However the reason for the decline in Mcl-1 itself has not been known.

Here we show that human neutrophils expressed only the cell cycle independent CDKs CDK 5, 7 and 9. These data are in broad agreement with previous reports showing that promyeloid progenitor cells lose expression of cell cycle dependent CDKs as they mature and differentiate towards neutrophils [7] and that neutrophils express cell cycle independent CDKs such as CDK5 [20]. The manuscript by Rossi *et al* [6] suggested that the pro-apoptotic effects of roscovitine were due to inhibition of CDK1 and CDK2, but did not consider the cell cycle independent CDKs. We could not detect CDK1 or CDK2 in our neutrophil preparations and it may be that differences in the purity of the preparations could explain this discordance as ours were >98% pure. What our data show is not only that the transcriptional cyclin-dependent kinase CDK9 is expressed in neutrophils but also that its activity declines as they age and enter apoptosis. Moreover, inhibition of CDK9 accelerated loss of Mcl-1 and induced rapid entry into apoptosis, but did not affect expression of Bcl-2A whose protein level did not decline as cells aged and entered apoptosis. We therefore propose that CDK9 activity ultimately determines the level of Mcl-1 and its loss is the cause of the very short lifespan of neutrophils.

Rather like peeling the layers from an onion, the next question is of course why CDK9 activity declines. Neutrophils are terminally differentiated cells, which have a characteristic multi-lobed nucleus, the result of the collapse of much of their chromatin. These cells thus have a limited capability for transcription and de novo protein synthesis and it was possible that CDK9 protein levels could not be maintained as neutrophils aged. The relative stability of CDK9 protein is in agreement with previous data showing that its expression was constant in HL60 cells through the cell cycle and also did not decline as these cells differentiated towards the monocyte lineage [21]. Further study revealed that the CDK9 co-factor cyclin T1 was lost as neutrophils aged in culture and this is therefore the likely cause of the loss of CDK9 activity [22]. Moreover, as cyclin T1 monomeric CDK9 has been proposed to be unstable and targeted for degradation [22] it is possible that CDK9 protein levels will eventually also fall once cyclin T1 is lost.

The importance of these data extends beyond neutrophil biology. Inhibition of neutrophil apoptosis is associated with chronic inflammatory disease such as Adult Respiratory Distress Syndrome [23] and Rheumatoid Arthritis [24] and the bactericidal functions of these cells are thought contribute significantly to tissue damage in these conditions. Inhibition of CDK9 resulting in the removal of neutrophils from sites of inflammation could represent a rational and novel therapeutic approach to the treatment of chronic inflammatory disease. Flavopiridol (Alvocidib) in clinical trial as a treatment for cancers including pancreatic cancer [25] multiple myeloma [26] and recently Sekine et al [27] showed that flavopiridol was effective at reducing joint inflammation in the collagen-induced arthritis model, but did not discover the cellular target. These authors assumed that the effect was likely to be mediated by inhibition of lymphocyte proliferation or function and when this was shown not to be the cases they did not look at other cells. Our data suggest that neutrophils were the likely cellular target of flavopiridol in their model and moreover that Alvocidib should be considered as a therapy for chronic inflammatory diseases.

## Materials and Methods

### Ethics statement

The study was approved by the Coventry and Warwickshire Research Ethics Committee (06/Q0201/2) and all subjects gave their written informed consent before taking part in the study.

### Isolation and culture of human neutrophils

Neutrophils were isolated from the peripheral blood of healthy volunteers as previously described [28]. Briefly, blood was collected into tubes containing EDTA and erythrocytes sedimented with 2% dextran T-500 in normal saline. Neutrophils were isolated using a discontinuous density gradient of Percoll (Sigma-Aldrich, Poole, UK). After hypotonic lysis to eliminate any remaining erythrocytes, neutrophils were resuspended in a round-bottom polypropylene tube in RPMI 1640 medium (Sigma-Aldrich) supplemented with 10% heat-inactivated fetal bovine serum (Sera Laboratories International, Bolney, UK) and containing 2 mM glutamine, 100 U/ml penicillin and 100ug/ml streptomycin (Sigma-Aldrich), termed complete medium. The neutrophils were >98% pure as determined by Giemsa differential staining and light microscopy and the only contaminating cells were eosinophils.

Neutrophils were cultured for up to 20h in the presence of a range of concentrations of the CDK inhibitors R-roscovitine or flavopiridol (Sigma Aldrich, Poole, UK). A novel roscovitine analogue LGR1406 [11] was a gift from Libor Havlíček (Institute of Experimental Botany ASCR, Prague) and NU6102 was kindly provided by D.R.Newell (University of Newcastle, UK).

### Measurement of Neutrophil Apoptosis

Neutrophil apoptosis was determined by observation of cell morphology by light microscopy following Giemsa staining [29] and assessment of loss of mitochondrial membrane integrity using uptake of DiOC_6_ (Invitrogen Ltd, Paisley, UK.) measured by flow cytometric analysis [30]. DiOC_6_ low cells were taken as apoptotic.

### Measurement of CDK9 activity

CDK9 activity in neutrophils was determined by immunoprecipitation of CDK9 from cells followed by an *in vitro* kinase assay. Briefly, neutrophils were resuspended in complete RPMI 1640 medium and incubated for 0 or 9 hours at 37°C, 5%CO2. Post incubation neutrophils were lysed in 1 ml lysis buffer (50 mM Tris, 150 mM NaCl, 4 mM AEBSF, 10 ug/ml leupeptin, 10 ug/ml pepstatin, 10 ug/ml aprotonin, 1% (v/v) Triton x-100, 2 mM NAF, 1 mM DTT, 1 mM EDTA, 1 uM Cathepsin inhibitor, 1 uM calpain inhibitor, 10% Glycerol, all purchased from Sigma-Aldrich) and left on ice for 20 minutes. Lysates were homogenised using a tight fitting dounce homogeniser followed by centrifugation at 10,000 xg for 10 minutes at 4°C. Supernatants were incubated with 2.5 ug/ml of anti-CDK9 antibody (Cell Signaling Technology, New England Biobs, UK) and 100 ul of protein G beads (Miltenyi Biotec, Bisley, UK) followed by incubation on ice for 30 minutes. Following incubation the CDK9 immunoprecipitate-bead complex was combined with 25 ul of kinase assay mix (50 mM Tris, 10 mM MgCl2, 1 mM DTT, protease inhibitor cocktail (Sigma Aldrich), phosphatase inhibitors I and II (Sigma Aldrich), 1 ug RNA polymerase II CTD repeat (AbCam, Cambridge MA, USA), 1 mM ATP and ^32^P-γATP (2.5 µCi, GE Healthcare Ltd, Amersham, UK)) and incubated at 30°C for 1 hour. The assay mixture was then applied to the magnetic bead isolation column (Miltenyi µ column, Miltenyi Biotec) and the eluate containing the labelled substrate was collected and transferred to nitrocellulose paper which was left to air dry. The nitrocellulose was washed 3 times using 1% (v/v) phosphoric acid and radioactivity (CPM) was then measured using a liquid scintillation counter.

### Measurement of CDK, cyclin T1, Bcl2A and Mcl-1 expression

Neutrophil total proteins were precipitated with 10% TCA, precipitates were washed in ice-cold acetone and resuspended in SDS-PAGE sample buffer before analysis for CDK, cyclin T1, Bcl2A and Mcl-1 content by Western blotting. Blots were probed with monoclonal antibodies to CDK1 and CDK2 (BD Transduction laboratories, Oxford, UK), CDK4, CDK5, CDK6, and CDK7 (Cell Signaling Technology), or CDK9, cyclin T1, Bcl2A and Mcl-1 (Santa Cruz Biotechnology) and isotype specific HRP-conjugated anti-mouse secondary antibodies (Dako cytomation Ltd, Ely, UK). Blots were visualised using enhanced chemiluminescence (GE Healthcare Ltd) and were probed with antibody to β-actin (Sigma-Aldrich) to confirm equal protein loading.

### Statistical analysis

Data were analysed by analysis of variance (ANOVA) followed by Student-Newman-Keuls comparison test. Data are expressed as mean ± s.d. and a p value of <0.05 was considered to indicate a significant difference between groups.
